# Induction of Monocyte Chemoattractant Proteins in Macrophages via the Production of Granulocyte/Macrophage Colony-Stimulating Factor by Breast Cancer Cells

**DOI:** 10.3389/fimmu.2016.00002

**Published:** 2016-01-20

**Authors:** Teizo Yoshimura, Tomozumi Imamichi, Jonathan M. Weiss, Miwa Sato, Liangzhu Li, Akihiro Matsukawa, Ji Ming Wang

**Affiliations:** ^1^Cancer and Inflammation Program, Center for Cancer Research, National Cancer Institute, Frederick, MD, USA; ^2^Department of Pathology and Experimental Medicine, Graduate School of Medicine, Dentistry and Pharmaceutical Sciences, Okayama University, Okayama, Japan; ^3^Laboratory of Human Retrovirology and Immunoinformatics, Leidos Biomedical Research, Inc., Frederick National Laboratory for Cancer Research, Frederick, MD, USA; ^4^Engineering Research Center for Cell and Therapeutic Antibody of Ministry of Education, School of Pharmacy, Shanghai Jiaotong University, Shanghai, China

**Keywords:** monocytes/macrophages, chemokines, inflammation, tumor microenvironment, breast cancer

## Abstract

Monocyte chemoattractant protein-1 (MCP-1)/CCL2 plays an important role in the initiation and progression of cancer. We previously reported that in 4T1 murine breast cancer, non-tumor stromal cells, including macrophages, were the major source of MCP-1. In the present study, we analyzed the potential mechanisms by which MCP-1 is upregulated in macrophages infiltrating 4T1 tumors. We found that cell-free culture supernatants of 4T1 cells (4T1-sup) markedly upregulated MCP-1 production by peritoneal inflammatory macrophages. 4T1-sup also upregulated other MCPs, such as MCP-3/CCL7 and MCP-5/CCL12, but modestly upregulated neutrophil chemotactic chemokines, such as KC/CXCL1 or MIP-2/CXCL2. Physicochemical analysis indicated that an approximately 2–3 kDa 4T1 cell product was responsible for the capacity of 4T1-sup to upregulate MCP-1 expression by macrophages. A neutralizing antibody against granulocyte/macrophage colony-stimulating factor (GM-CSF), but not macrophage CSF, almost completely abrogated MCP-1-inducing activity of 4T1-sup, and recombinant GM-CSF potently upregulated MCP-1 production by macrophages. The expression levels of GM-CSF in 4T1 tumors *in vivo* were higher than other tumors, such as Lewis lung carcinoma. Treatment of mice with anti-GM-CSF antibody significantly reduced the growth of 4T1 tumors at the injection sites but did not reduce MCP-1 production or lung metastasis in tumor-bearing mice. These results indicate that 4T1 cells have the capacity to directly upregulate MCP-1 production by macrophages by releasing GM-CSF; however, other mechanisms are also involved in increased MCP-1 levels in the 4T1 tumor microenvironment.

## Introduction

Infiltration of leukocytes is observed in a number of human and mouse cancers (1, 2). Although the composition of tumor-infiltrating leukocytes and the role they play may vary in each tumor, they are generally immunosuppressive and provide a microenvironment that favors tumor growth. Thus, identifying the molecular mechanisms by which immunosuppressive leukocytes are recruited into tumors is critical and clinically important.

Monocyte chemoattractant protein-1 (MCP-1)/CCL2 is a chemokine with potent monocyte chemotactic activity. It was initially purified from the culture supernatant of a human malignant glioma (3) and a monocytic leukemic cell line (4), and is identical to the tumor-derived chemotactic factor, TDCF (5). Accumulating evidence strongly suggests that the production of MCP-1 by tumors is responsible for the recruitment of immunosuppressive macrophages that promote tumor growth. In a chemically induced skin papilloma model, the number of papillomas in MCP-1-deficient mice was lower compared to that observed in WT mice (6). A vital role of MCP-1 in the initiation and progression of colitis-associated colon carcinogenesis was demonstrated by using mice deficient in the MCP-1 receptor CCR2 or MCP-1 blocking agent (7). In addition, neutralization of MCP-1 resulted in reduced growth and development of prostate cancer (8–10), breast cancer (11, 12), and lung cancer (13) in mice. Thus, MCP-1 is a candidate molecular target of cancer treatment (14).

4T1 breast cancer was a spontaneous mammary tumor of a Balb/cC3H mouse. When 4T1 cells are orthotopically transplanted into mammary pads of Balb/c mice, they form tumors and metastasize to distant organs and tissues, such as lung, liver, and bone; thus, providing an excellent model to elucidate the mechanisms involved in tumor growth and metastasis (15). We previously reported that non-tumor stromal cells, but not tumor cells, were the main source of MCP-1 in 4T1 tumors, and stromal cell-derived MCP-1 played a critical role in the spontaneous metastasis of tumor cells to the lung (16).

In the present study, we examined the molecular mechanisms by which stromal cells, especially infiltrating macrophages, produce MCP-1 in 4T1 tumors. We identified granulocyte/macrophage colony-stimulating factor (GM-CSF/CSF-2) released by 4T1 cells as a potent inducer of monocyte chemotactic chemokines, including MCP-1, MCP-3, and MCP-5, by inflammatory mouse macrophages *in vitro*. Inhibition of GM-CSF by a neutralizing antibody *in vivo* significantly reduced tumor size, but not MCP-1 production or lung metastasis. These results indicate that tumor cell-derived GM-CSF promotes tumor progression by tuning the tumor-promoting microenvironment by activating tumor-infiltrating macrophages, but other mechanisms are also involved in increased MCP-1 production in the 4T1 tumor microenvironment. Better understanding of the interaction between tumors cells and non-tumor cells in tumor stroma may lead to the development of novel cancer treatment strategies.

## Materials and Methods

### Reagents

RPMI-1640, DMEM, HBSS, and Ultradoma were from Lonza, Walkersville, MD, USA. TRIzol reagent was from Invitrogen, Grand Island, NY, USA. Fetal bovine serum (FBS) was from HyClone, Logan, UT, USA. Recombinant mouse TNFα, M-CSF, GM-CSF, normal rat IgG, and neutralizing antibodies against mouse TNFα (clone MP6-XT22), mouse M-CSF (clone 131621), or mouse GM-CSF (clone MP122E9) were from R&D Systems, Minneapolis, MN, USA. Anti-mouse GM-CSF (clone MP122E9, LEAF™ purified) was also from BioLegend (San Diego, CA, USA). Recombinant human M-CSF and GM-CSF were from Peprotech, Rocky Hill, NJ, USA. Thioglycollate (TG) was from Difco Laboratories (Detroit, MI, USA). LPS was from Sigma-Aldrich, St. Louis, MO, USA. [α-^32^P]dCTP was from Perkin Elmer, Cambridge, MA, USA.

### Mice

Wild type (WT) C57BL/6 and Balb/c mice were from Charles River, Frederick, MD, USA. The generation of Balb/c MCP-1^−/−^ mice (MMRRC stock No. 037094-UNCC, 29S1(Cg)-Ccl2tm1.1Tyos/Mmnc) was previously described (16, 17). Myeloid cell-specific MCP-1^−/−^ mice were generated by crossing MCP-1^flox/flox^ mice (JAX Stock No. 023347, B6;129-Ccl2 <tm1Tyos>/J) (17, 18) to LysMCre mice (19). MyD88^−/−^, TLR2^−/−^, TLR4^−/−^, TLR9^−/−^, and IL-1R1^−/−^ mice on a C57BL/6 background were from the Cancer and Inflammation Program Mouse Core, NCI, Frederick. Mouse resident peritoneal cells (PC) were obtained by flushing the peritoneal cavity of C57BL/6 mouse with 5 ml clod PBS. Mouse peritoneal exudate cells (PEC) were induced by intraperitoneal injection of 3% TG and harvested 3–4 days later. The experimental protocols of this study were approved by the Frederick National Laboratory for Cancer Research Animal Care and Use Committee, Frederick, MD, USA.

### Generation of 4T1 Cell Culture Supernatant

Murine breast cancer 4T1 cells (ATCC, Manassas, VA, USA) were grown in RPMI-1640 containing 10% FBS, 1 mM glutamine, and antibiotics. Cell-free supernatants were collected by centrifugation at 1,200 rpm for 10 min. 4T1 cells were also grown in Ultradoma protein-free medium supplemented with 1% FBS, and cell-free culture supernatant was collected as described previously. Cell-free supernatants were concentrated by using Amicon Centriprep 10 or 30 (Amicon, Billerica, MA, USA).

### Column Chromatography

Thirty milliliter of 4T1-sup containing 1% FBS was concentrated by CentriPrep (molecular weight cut off 3,000, EMD Millipore, Billerica, MA, USA) to 0.5 ml, filtered, and injected into a TSK-2500 column (Tosoh Bioscience, King of Prussia, PA, USA). Four hundred microliter fractions were collected and assayed for MCP-1-inducing activity at 1:10 dilution.

### Northern Blotting

Northern blot analysis was performed as described in 1.2% agarose gels containing formaldehyde (20). Filters were prehybridized in Ultrahybri solution (Invitrogen) at 42°C for 1 h and then hybridized overnight in the presence of l × 10^6^ dpm/ml of ^32^P-labeled cDNA probe. Filters were washed twice with 2× SSC, 0.1% SDS at 42°C for 10 min and 0.1× SSC, 0.1% SDS at 60°C for 30 min prior to autoradiographic exposure. The cloning of mouse MCP-1, MCP-3, and MCP-5 cDNA and human MCP-1 and interleukin-8 (IL-8) was previously reported (17, 21, 22).

### Quantification of Cytokine/Chemokine Concentration

Mouse blood was drawn by heart puncture, and sera were isolated and stored at −70°C until use. The concentration of MCP-1 was measured in the Lymphokine Testing Laboratory, Clinical Services Program, SAIC-Frederick, Frederick, MD, USA or in our laboratory with an ELISA kit specific for mouse MCP-1 (R&D Systems). Cytokine levels in mouse serum and cell supernatant samples were analyzed using a Cytometric Bead Array Flex Set according to the manufacturer’s instructions on an LSR-II flow cytometer (BD Biosciences, Mountain View, CA, USA).

To quantitate MCP-1 levels in tumor tissues, tumors were homogenized in RIPA lysis buffer containing protease inhibitors. The homogenates were spun, and the supernatants were frozen at −20°C until use. The concentration of MCP-1 was analyzed by ELISA (R&D Systems). The concentration of total protein contained in the tumor lysates was determined by BCA protein assay kit (Pierce). The results are presented as picogram MCP-1/milligram total protein in the sample.

### Activation of Macrophages *In Vitro*

Peritoneal exudate cells (PEC) were harvested by peritoneal lavage, using 5 ml cold PBS. After centrifugation, PEC were resuspended in RPMI-1640 containing 10% FBS, penicillin, and streptomycin, at the concentration of 2.5 × 10^6^, and then cultured for indicated times in the presence or absence of stimuli.

To prepare human macrophages, 2 × 10^6^ human monocytes were cultured in a six-well plate in 4 ml RPMI1640 containing 10% FBS and 50 ng/ml recombinant human M-CSF for 7 days. Cells were incubated overnight in RPMI1640 without serum and then activated with 100 ng/ml LPS or different concentrations of recombinant human GM-CSF for 5 h. Total RNA was isolated and subjected to Northern blotting or quantative PCR (qPCR).

### Gene Expression Profiling

Total RNA was extracted by TRIzol (Invitrogen), and the profile of gene expression was analyzed using the nCounter Analysis System (NanoString Technologies, Seattle, WA, USA) (23). The nCounter Code Set for the study contained 41 test and 2 control genes. The assay used two sequence-specific probes for each gene of interest. The probes were complementary to a 100-base region of the target mRNA. One probe was covalently linked to an oligonucleotide containing biotin (the capture probe); the other was linked to a color-coded molecular tag (reporter probe). Each hybridization consisted of 100 ng of total RNA, reporter, and capture probe mix for the 43 genes. The hybridization, washing, and scanning procedures were conducted according to the guidelines provided by NanoString Technologies.

### qRT-PCR

The expression of 47 cytokine and 28 chemokine genes was assessed by qRT-PCR. Total RNA was extracted from non-activated or activated PEC by using RNeasy mini kit (Qiagen). The quality and yield were assessed by Nanodrop spectrophotometry, and then cDNA was synthesized using High Capacity cDNA Reverse Transcription Kit (Applied Biosystems). Real-time PCR was run on a Step One Plus Real-time PCR system (Applied Biosystems) using Power SYBR Green PCR Master Mix (Applied Biosystems) and Mouse Cytokine Primer Library I and II (RealTimePrimers.com). The cycling condition was as follows: 10 min incubation at 95°C, followed by 50 cycles of 95°C for 15 s, and 60°C for 1 min. The specificity of the PCR products was examined by a final melting curve analysis (95°C for 15 s, 60°C for 1 min, and 95°C for 15 s). The fold expression or repression of the target gene relative to the internal control gene, β-actin, in each sample was calculated.

### Treatment of Mice with Anti-GM-CSF Antibody

4T1 cells were grown to 50–80% confluence. Before injection, cells were detached with 0.2% trypsin–EDTA, washed once with medium, three times with PBS, and resuspended in PBS at 1 × 10^6^ cells/ml. One hundred microliter of cell suspension (1 × 10^5^ cells) were injected into the second mammary pad of six female Balb/c mice. Mice were separated into two groups; three mice in the control group received intraperitoneal injection of normal rat IgG (100 μg in 100 μl PBS) whereas three mice in the experiment group received anti-mouse GM-CSF IgG (100 μg in 100 μl PBS) twice a week, for 3 weeks. Blood was collected by mandibular puncture on day 14 and by heart puncture on day 28, and sera were isolated and stored at −80°C until use. Tumors and spleens were excised on day 28, and the infiltration of myeloid-derived suppressor cells (MDSCs) and macrophages was evaluated by flow cytometry. Lungs were perfused with Bouin’s solution, fixed in the same solution, and then the number of tumor nodules was counted by eye. In a separate experiment, 1 × 10^5^ 4T1 cells (100 μl) were mixed with 10 μg of normal rat IgG or anti-GM-CSF IgG and injected into the second mammary pad of WT mice. Mice then received intra-tumor injection of 20 μg of normal rat IgG or anti-GM-CSF IgG on day 5 and 9, followed by 40 μg of normal rat IgG or anti-GM-CSF IgG on day 12. Blood was collected by mandibular puncture on day 14, and sera were isolated and stored at −80°C until use. Tumor size was measured, and tumor volume was calculated using the following formula: volume = (width)^2^ × length/2.

### Isolation of Tumor-Infiltrating Leukocytes

Tumors were dissected on day 28, homogenized using a GentleMACS dissociator (Miltenyi Biotech) and digested in RPMI containing 5% FBS, 250 U/ml type IV collagenase (Invitrogen), 100 μg/ml DNase I (Roche Molecular Biochemicals), and 1 mM EDTA (pH 8.0), at 37°C for 20 min. The homogenate was then processed in a tissue stomacher-80 (Seward) for 30 s, washed with HBSS, and resuspended in 40% Percoll (Amersham Pharmacia) in DMEM. The suspension was underlaid with 80% Percoll and centrifuged for 25 min at 1,000 × *g*. Leukocytes were collected from the interphase, washed, counted, and stained with anti-mouse antibodies, including FITC-CD45, APC-CD11b, PerCPCy5.5-Gr1, PerCPCy5.5-CD19, APC-CD8 (BD Biosciences), PE-F4/80, APC eFluor 780-CD4, PE-CD25, and Pacific Blue-NKp46 (eBiosciences). Acquisition of data was performed using a LSRII, and data analysis was conducted using the FlowJo software (Tree Star Inc., Ashland, OR, USA). Only live cells determined using Sytox blue live/dead stain (Life Technologies) were analyzed.

### Statistical Analysis

All experiments were performed at least twice. Results presented are from representative experiments. Statistical analysis was performed by Student’s *t*-test, using the GraphPad Prism, Version 4 and 5, GraphPad Software, San Diego, CA, USA. A value of *p* < 0.05 was considered to be statistically significant.

## Results

### 4T1 Cell Product(s) Induces the Expression of MCP-1 and Other MCPs in Inflammatory Macrophages

**Figure 1:**
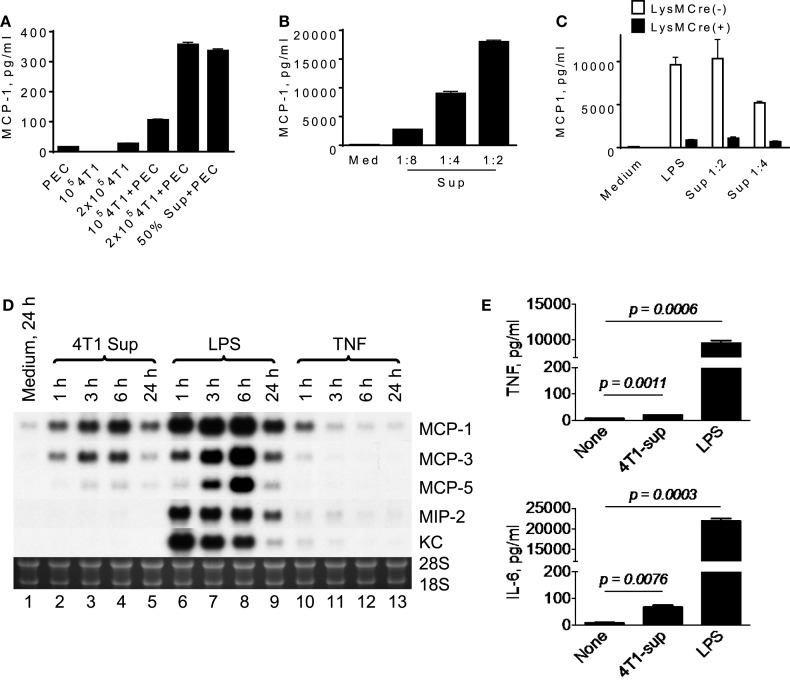
**A product of 4T1 cells induces MCP-1 production by macrophages**. **(A)** 1 × 10^5^ or 2 × 10^5^ 4T1 cells were seeded into six-well plates. After overnight incubation at 37°C, medium was replaced by fresh medium, and 2.5 × 10^6^ TG-induced PEC (Balb/c) or an equal volume of 4T1-sup were added. After 24 h-incubation, cell-free culture supernatants were collected, and MCP-1 concentration in each supernatant was measured by MCP-1 ELISA. The results are shown as the mean ± SD, *n* = 3. **(B)** Five million TG-induced PEC were cultured in the presence of serially diluted 4T1-sup. After 24 h-incubation, cell-free media were collected, and MCP-1 concentration in each medium was measured by MCP-1 ELISA. The results are shown as the mean ± SD, *n* = 3. **(C)** Five million TG-induced PEC from MCP-1^flox/flox^ or LysMCre^+^/MCP-1^flox/flox^ mice were incubated in the presence of 100 ng/ml LPS or 4T1 supernatant. After 24 h-incubation, cell-free media were collected, and MCP-1 concentration in each medium was measured by MCP-1 ELISA. The results are shown as the mean ± SD, *n* = 3. **(D)** Two and half million TG-induced PEC were incubated in the presence or absence of 4T1-sup (1:1), 100 ng/ml LPS, or 10 ng/ml TNFα. Total RNA was extracted and subjected to Northern blotting. **(E)** Two million TG-induced PEC were cultured in the presence of 4T1-sup (1:1). After 24 h-incubation, cell-free media were collected, and TNFα or IL-6 concentration in each medium was measured by Cytometric Bead Array. The results are shown as the mean ± SD, *n* = 2.

To analyze the mechanisms by which infiltrating macrophages produce MCP-1 in 4T1 tumors, we first incubated mouse TG-induced PEC with tumor cells or with the culture supernatant of tumor cells (4T1-sup) for 24 h and measured the concentration of MCP-1 in the supernatants. As shown in Figure 1A, a low level of MCP-1 was detected in the culture supernatant of unstimulated TG-induced PEC (2.5 × 10^6^ cells/ml). There was no detectable level of MCP-1 in the culture supernatant of 1 × 10^5^ 4T1 cells, but a low level of MCP-1 could be detected when the cell number was increased to 2 × 10^5^. When PEC were co-cultured with 4T1 cells or cultured in the presence of 50% (*v*/*v*) 4T1-sup, the level of MCP-1 in the culture supernatants was markedly increased in a manner dependent on the number of 4T1 cells (Figure 1A) or the concentration of 4T1-sup (Figure 1B). 4T1-sup did not increase the production of MCP-1 by TG-induced PEC from mice in which the MCP-1 gene was specifically deleted in myeloid cells, confirming that myeloid cells, especially macrophages, were the source of MCP-1 in the culture (Figure 1C).

To examine whether the effect of 4T1-sup was selective to MCP-1, we examined the expression of several chemokines, including MCP-3/CCL7, MCP-5/CCL12, MIP-2/CXCL2, and KC/CXCL1 by PEC, by Northern blotting. As shown in Figure 1D, 4T1-sup induced a high level of MCP-3 and a low level of MCP-5 mRNA, but the induction of neutrophil-attracting chemokines MIP-2 or KC mRNA appeared to be minimal (lanes 2–5). In contrast, the TLR4 ligand LPS strongly induced the upregulation of all chemokines in PEC (lanes 6–9). TNFα induced only a low level of MCP-1 at 1 h (lanes 10–13). We also examined the production of tumor-promoting cytokines, such as TNFα or interleukin-6, by 4T1-sup-activated PEC (Figure 1E). There was a significant increase in the concentration of these cytokines in the culture supernatant, but the increase was much smaller than that by LPS. These results indicated that 4T1 cells have the capacity to activate macrophages to produce chemokines that recruit monocytes to shape the tumor microenvironment.

### 4T1 Sup-Induced MCP-1 Production by Macrophages Was Independent of TNFα, TLR, or IL-1R1 Agonists

**Figure 2:**
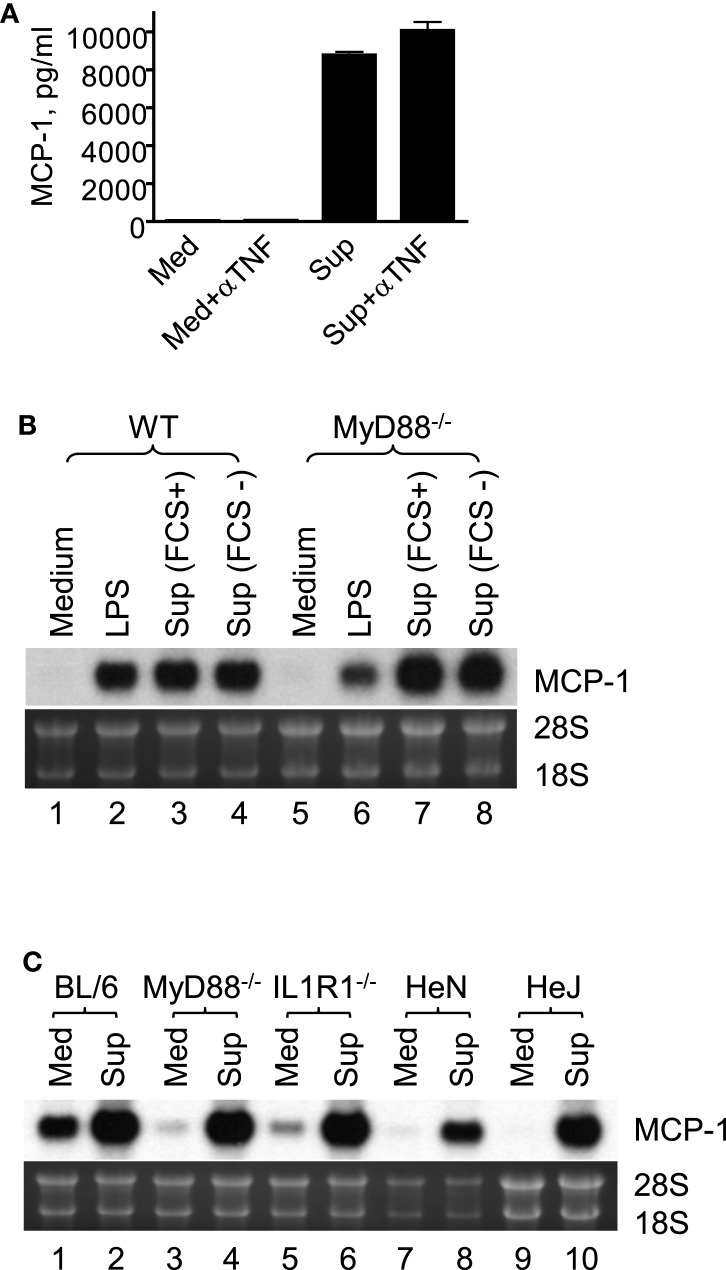
**Macrophage production of MCP-1 in response to stimulation by 4T1 supernatant is independent of TNFα, IL-1R1, TLR4, or MyD88**. **(A)** Five million WT TG-induced PEC were incubated in the presence or absence of 4T1-sup (1:1) or 10 ng/ml TNFα for 24 h and the concentration of MCP-1 in cell-free culture supernatants was measured by ELISA. To neutralize TNFα, 10 μg neutralizing anti-TNFα IgG was added to the culture. The results are shown as the mean ± SD, *n* = 2. **(B)** Two and half million TG-induced PEC from WT or MyD88^−/−^ mice were cultured for 4 h in the presence of 100 ng/ml LPS or 4T1-sup. Total RNA was isolated and subjected to Northern blotting. **(C)** Two and half million TG-induced PEC from WT or MyD88^−/−^, IL-1R1^−/−^, C3H/HeN (TLR4^+/+^), or C3H/HeJ (TLR4^−/−^) mice were cultured for 4 h in the presence of 4T1-sup (1:1). Total RNA was isolated and subjected to Northern blotting.

It is well known that tumor cells release proinflammatory mediators, such as TNFα (24), IL-1 (25), and TLR agonists (26), which activate macrophages. Therefore, we examined whether MCP-1 production induced by 4T1 sup was due to these proinflammatory mediators by ELISA or Northern blotting. As shown in Figure 2A, addition of neutralizing antibody against TNFα had no effect on macrophage MCP-1 production induced by 4T1-sup. PEC deficient of MyD88, IL-1 R1, or TLR4 (HeJ mice) expressed MCP-1 mRNA whose level was comparable to that expressed by WT PEC (Figures 2B,C). These results indicated that 4T1-sup-induced MCP-1 production by macrophages was not dependent on TNFα, IL-1, or TLR agonists that use MyD88 to transduce intracellular signals. Thus, 4T1 cells release other mediator(s) to activate macrophages for MCP-1 production.

### GM-CSF in 4T1-Sup Was Responsible for 4T1-Sup-Induced MCP-1 Expression by Macrophages

**Figure 3:**
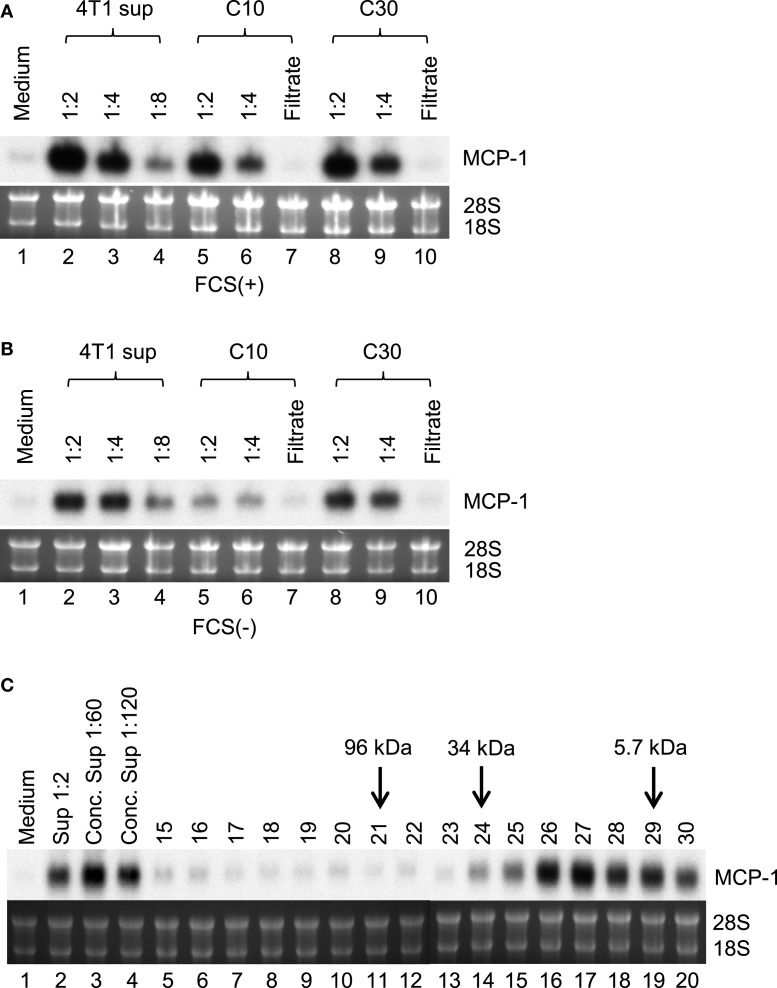
**Partial characterization of MCP-1-inducing activity in 4T1-sup**. Cell-free 4T1-sup containing 10% FBS **(A)** or without FBS **(B)** was concentrated by using Amicon Centriprep 10 or 30, and then total volume was adjusted to the original volume by adding RPMI-1640. MCP-1-inducing activity was examined by Northern blotting. **(C)** Thirty milliliter of 4T1-sup containing 1% FBS was concentrated by CentriPrep, filtered, and injected into a TSK-2500 column. Four hundred micro liter fractions were collected and assayed for MCP-1-inducing activity at 1:10 dilution. The fractions to which molecular mass markers were eluted are indicated by arrows.

To identify the 4T1 cell-derived molecule that activates macrophages, we first attempted to determine its molecular mass. We prepared 4T1-sup in the absence or presence of 10% FBS and concentrated them on an Amicon membrane with a molecular mass cutoff of 10 (C10) or 30 kDa (C30). As shown in Figures 3A,B, the majority of MCP-1-inducing activity was retained on either filter, suggesting that the molecular mass of this molecule was larger than 30 kDa. Molecular sieve column chromatography showed a peak MCP-1-inducing activity in the fraction 26 and 27, which corresponded to the molecular mass of 20–26 kDa (Figure 3C).

**Figure 4:**
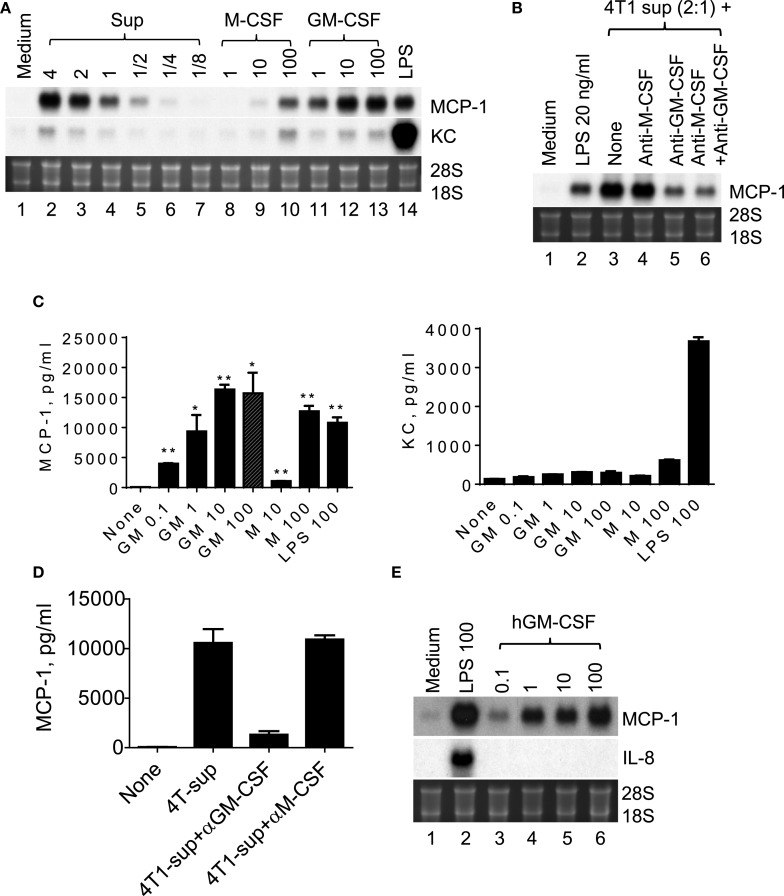
**GM-CSF produced by 4T1 cells is responsible for the MCP-1 expression by macrophages**. **(A)** Two and half million TG-induced PEC from WT mice were cultured for 4 h in the presence of different concentration of 4T1-sup, mouse recombinant M-CSF or GM-CSF for 4 h. Total RNA was isolated and subjected to Northern blotting. **(B)** Two and half million TG-induced PEC from WT mice were cultured for 4 h with 4T1-sup in the presence or absence of neutralizing antibody against mouse M-CSF or GM-CSF for 4 h. Total RNA was isolated and subjected to Northern blotting. **(C)** Two million TG-induced PEC from WT mice were cultured for 24 h in the presence of different concentrations of recombinant mouse GM-CSF, M-CSF, or LPS. Cell-free culture supernatants were obtained, and the concentration of MCP-1 or KC was measured by ELISA for MCP-1 and by Cytometric Bead Array for KC. The results are shown as the mean ± SD, *n* = 2. **(D)** Two million TG-induced PEC from WT mice were cultured for 24 h with 4T1-sup in the presence or absence of neutralizing antibody against mouse M-CSF or GM-CSF. Cell-free culture supernatants were obtained and the concentration of MCP-1 was measured by ELISA. The results are shown as the mean ± SD, *n* = 2. **(E)** Human monocyte-derived macrophages were cultured with 100 ng/ml LPS or different concentrations of recombinant human GM-CSG for 4 h. Total RNA was isolated and subjected to Northern blotting.

4T1 cells were previously shown to express transcripts for myeloid cell growth factors, such as M-CSF/CSF-1, GM-CSF/CSF-2, and G-CSF/CSF-3, and M-CSF was able to induce MCP-1 production in macrophages (27), suggesting that M-CSF produced by 4T1 cells may be responsible for the increased MCP-1 production by macrophages. Therefore, we examined the capacity of recombinant mouse M-CSF to induce MCP-1 expression by macrophages. As shown in Figure 4A, 4T1-sup dose-dependently induced high levels of MCP-1 mRNA expression in PEC (lanes 2–7). M-CSF induced moderate levels of MCP-1 mRNA expression at its highest concentration of 100 ng/ml (lanes 8–10). In contrast, GM-CSF increased MCP-1 expression by PEC at as low as 1 ng/ml and its peak activity was detected at 10 ng/ml; thus GM-CSF is a potent inducer of MCP-1 in macrophages. Similar to 4T1-sup, recombinant GM-CSF induced a high level of MCP-1 mRNA, but not KC mRNA, by Northern blotting (Figure 4A).

**Figure 5:**
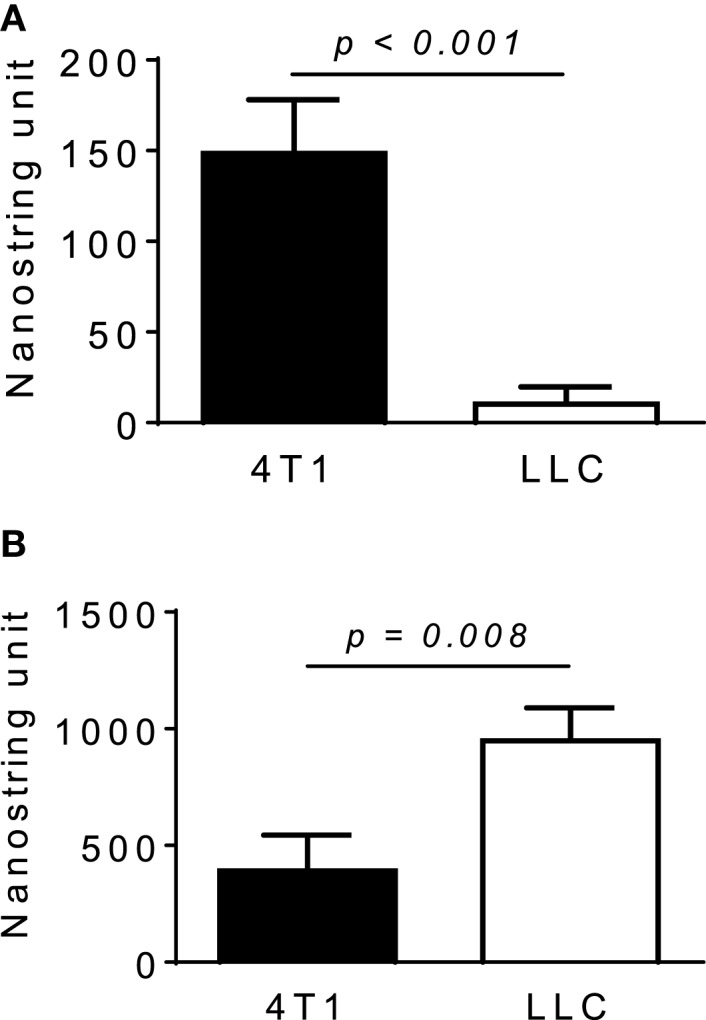
**Expression of M-CSF or GM-CSF in 4T1 or LLC tumors**. The expression of GM-CSF **(A)** or M-CSF **(B)** mRNA in 4T1 tumors or LLC tumors was examined by Nanostring gene profiling. The results are shown as the mean ± SEM, *n* = 5 for 4T1 tumors and *n* = 4 for LLC tumors.

We next used neutralizing antibodies and found that anti-GM-CSF Ab, but not anti-M-CSF Ab, almost completely abrogated 4T1-sup-induced MCP-1 expression by PEC (Figure 4B). There was no additional inhibition when both antibodies were used together. The results of MCP-1 mRNA expression were validated by the results of MCP-1 protein production (Figures 4C,D). Human recombinant GM-CSF also dose-dependently induced high levels of MCP-1, but not IL-8, expression in human monocyte-derived macrophages (Figure 4E). Thus, GM-CSF is a potent inducer of MCP-1 in macrophages, and GM-CSF produced by breast cancer cells may contribute to the elevated MCP-1 production in tumor stroma. In fact, GM-CSF was highly expressed in 4T1 tumors (Figure 5A), but not in tumors generated by the subcutaneous injection of the mouse lung cancer LLC cells. In contrast, M-CSF was more highly expressed in LLC tumors than in 4T1 tumors (Figure 5B).

The results of Northern blotting shown above suggested that GM-CSF, unlike LPS that potently induces a wide variety of cytokine and chemokine genes by macrophages, might induce a selective set of cytokine and chemokine genes. Therefore, we examined the expression of 47 cytokine and 28 chemokine genes by GM-CSF-activated mouse macrophages by qRT-PCR and compared with that by LPS-activated macrophages. GM-CSF highly induced the expression of several cytokine genes, including TNFα, TNF subfamily member 11 (Trance/RankL), IL-1 family member 8 (IL-36B), and chemokine genes, including CXCL1, CXCL2, CXCL5, CCL2, CCL3, CCL7, CCL17, and CCL28. However, the expression of TNFα, CXCL1 (KC), and CXCL2 (MIP-2) was much lower than that induced by LPS. The expression of other genes highly induced by LPS, such as IL-1α, IL-1β, IL-1 member 6, IL-6, IL-17F, CXCL12, and CCL5, was also low in GM-CSF-activated macrophages. Thus, GM-CSF induces the expression of a set of cytokine and chemokine genes that are different from those induced by LPS in mouse macrophages (Table S1 in Supplementary Material).

### Neutralization of GM-CSF Did Not Reduce either MCP-1 Production or Lung Metastasis in Tumor-Bearing Mice

The results of our *in vitro* study suggested that GM-CSF secreted by tumor cells regulate the production of MCP-1 in 4T1 tumors; therefore, neutralization of GM-CSF in tumor-bearing mice may reduce MCP-1 production in 4T1 tumor microenvironment and subsequent lung metastasis of 4T1 cells. We first examined whether neutralization of GM-CSF directly affects the growth of 4T1 cells *in vitro*. Ten thousands 4T1 cells were seeded in six-well culture plates and incubated at 37°C for 4 or 5 days in the presence of normal rat IgG or anti-GM-CSF Ab. Addition of anti-GM-CSF Ab had no direct effect on the growth of 4T1 cells (data not shown).

**Figure 6:**
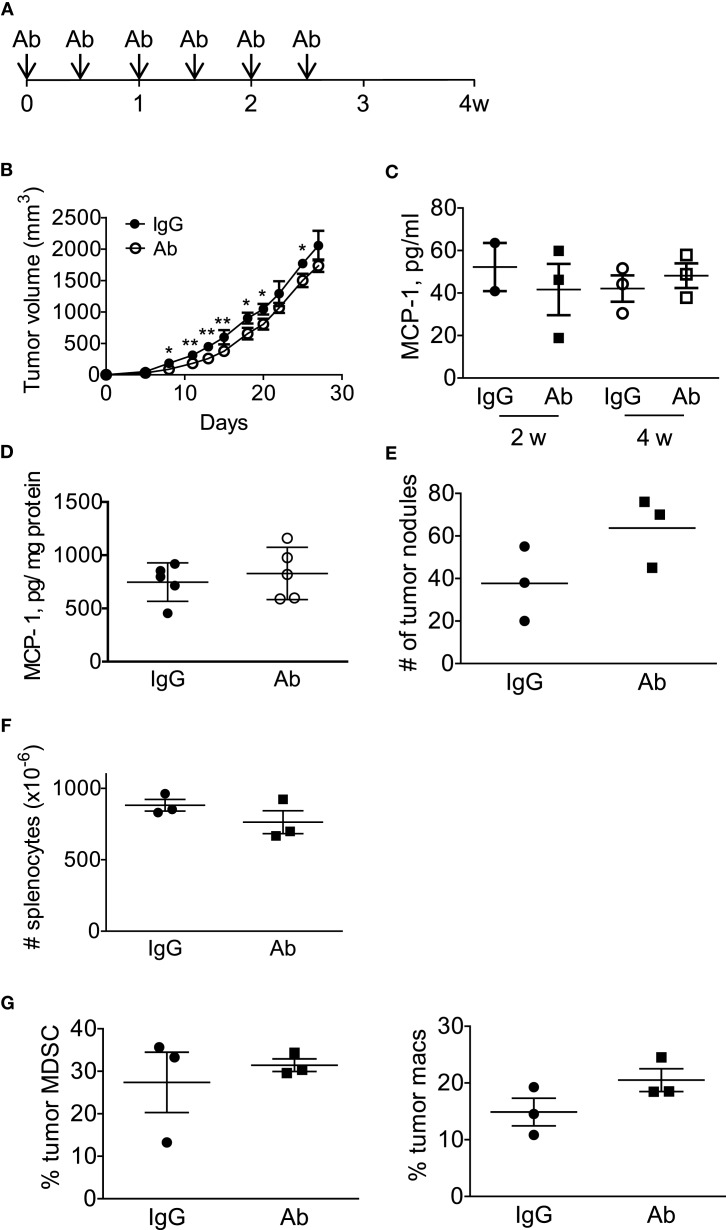
**Anti-GM-CSF treatment of 4T1 tumor-bearing mice had a limited effect on tumor progression**. **(A)** The scheme of systemic antibody treatment is shown. One hundred micro gram of either control rat IgG or anti-GM-CSF Ab was intraperitoneally injected on day 0, 3, 7, 10, 14, 18. Mice were euthanized on day 28. **(B)** The size of tumor in each mouse was measured, and the volume was calculated. The results are shown as the mean ± SD, *n* = 3, *p < *0.05*, **p < *0.01*. **(C)** Serum samples were collected on day 14 and day 28, and the MCP-1 concentration was measured by ELISA. The results are shown as the mean ± SD, *n* = 3. **(D)** Tumors were homogenized in RIPA lysis buffer-containing protease inhibitors and the homogenates were spun and the supernatants were frozen at −20°C until use. The concentration of MCP-1 was analyzed by ELISA, and the concentration of total protein in the tumor lysates was determined by BCA protein assay kit. The results are shown as the mean ± SD, *n* = 5. **(E)** The number of tumor nodules on the lung of each mouse was counted on day 28. The results are shown as the mean ± SD, *n* = 3. **(F)** The number of splenocytes of each mouse was counted on day 28. The results are shown as the mean ± SD, *n* = 3. **(G)** The percentage of tumor-infiltrating CD11b^+^Gr1^low^F4/80^−^ MDSC or F4/80^+^ macrophages in CD45^+^ cells was analyzed by flow cytometry. The number of tumor nodules on the lung of each mouse was counted on day 28. The results are shown as the mean ± SD, *n* = 3.

**Figure 7:**
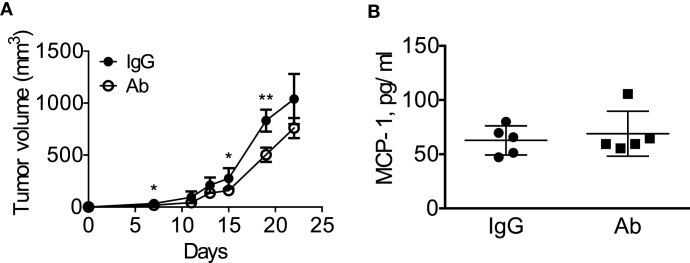
**Intratumoral injection of anti-GM-CSF had a limited effect on tumor progression in 4T1 tumor-bearing mice**. **(A)** 1 × 10^5^ 4T1 cells (100 μl) were mixed with 10 μg of normal rat IgG or anti-GM–CSF IgG and injected into the second mammary pad of WT mice. Mice were then received intra-tumor injection of 20 μg of normal rat IgG or anti-GM-CSF IgG on day 5 and 9, followed by 40 μg of normal rat IgG or anti-GM-CSF IgG on day 12, 16, and 19. The size of tumor in each mouse was measured and the volume was calculated. The results are shown as the mean ± SD, *n* = 5, *p < 0.05, **p < 0.01. **(B)** Blood was collected by mandibular puncture on day 14, sera were isolated, and MCP-1 concentration was measured by ELISA. The results are shown as the mean ± SEM, *n* = 5.

We next treated the mice with intraperitoneal injection of either normal rat IgG or anti-GM-CSF Ab (100 μg per mouse) six times (Figure 6A) to examine the role of tumor-cell-derived GM-CSF in MCP-1 production and tumor progression. As shown in Figure 6B, injection of anti-GM-CSF Ab significantly delayed the growth of primary tumors at an early stage, especially from day 11 to 15, but this effect became less significant at a later stage (after day 19). Either serum MCP-1 concentration or tumor MCP-1 content was not significantly different between the two mouse groups (Figures 6C,D), and mice treated with anti-GM-CSF Ab developed lung metastasis similar to those treated with normal rat IgG (Figure 6E). Enlargement of spleens was observed in both group with similar numbers of splenocytes (Figure 6F). Furthermore, the percentage of MDSCs and macrophages infiltrating the primary tumors was similar between the two groups (Figure 6G). The distribution of macrophages in tumors was also similar between the two groups (data not shown). Intratumoral injection of anti-GM-CSF Ab also significantly reduced tumor volume on day 7, 15, and 19, but did not reduce the serum MCP-1 concentration 2 weeks after the injection of tumor cells (Figures 7A,B). These results indicate that tumor cell-derived GM-CSF appears to play a role in promoting tumor development, but it is unlikely a major factor involved in MCP-1 production in 4T1 tumor microenvironment *in vivo*.

## Discussion

Tumor microenvironment consists of a variety of cell types, including tumor cells, fibroblasts, endothelial cells, myocytes, and inflammatory cells, such as MDSCs, regulatory T cells, macrophages, and dendritic cells. The interaction of tumor cells with stromal cells leads to the production of an array of mediators that provide the soil for tumor cells to grow, invade, and metastasize (28–30). We previously reported that the chemokine MCP-1 produced by non-tumor cells in tumor stroma, both hematopoietic and non-hematopoietic cells, promoted spontaneous lung metastasis of 4T1 breast cancer cells (16). However, the mechanisms by which stromal cells in 4T1 tumor microenvironment produce MCP-1 remain unclear. In the present study, we examined whether 4T1 cells directly activate inflammatory macrophages, an important cellular component of tumor stroma, and found that GM-CSF produced and released by 4T1 cells potently induces MCP-1 production by mouse inflammatory macrophages.

In breast cancer, macrophages constitute up to 35% of the infiltrating inflammatory cells (31), and the behavior of macrophages is conditioned by cytokines produced in a tumor microenvironment. The macrophage growth factor M-CSF is expressed in over 70% of human breast cancers (32). Serum M-CSF levels correlate with tumor size, metastasis, and poor outcomes in humans (33, 34). Mice deficient in M-CSF are protected against breast tumor metastasis, and re-expressing M-CSF solely in the breast tissue restores tumor metastatic capacity (35). M-CSF was shown to induce leukocytes to produce cytokines, including IL-1β and TNFα, and chemokines, including MCP-1 (36). These findings led us to hypothesize that M-CSF might be responsible for 4T1-sup-induced MCP-1 production by macrophages. Although the expression of M-CSF was detected in 4T1 tumor microenvironment, M-CSF was a weak inducer of MCP-1 in mouse inflammatory macrophages *in vitro*. Furthermore, neutralization of M-CSF did not affect the activity of 4T1-sup to induce MCP-1 production. These results indicate that M-CSF does not play a major role in macrophage expression of MCP-1 in 4T1 breast cancer.

Granulocyte/macrophage colony-stimulating factor, another macrophage growth factor, is produced by a wide variety of cells, including cancer cells (37). Recently, mesenchymal-like human breast cancer cells were demonstrated to produce GM-CSF and cancer cell-derived GM-CSF transformed macrophages to a TAM-like phenotype and to produce the chemokine CCL18, leading to the promotion of metastasis to the lung and liver in humanized mice (38). GM-CSF was highly expressed in about 29% of human breast cancer samples, and the infiltration of CCL18-positive macrophages was often observed at the invasive front of tumors, where cancer cells displayed more mesenchymal features and expressed high levels of GM-CSF. These finding led the authors to the conclusion that GM-CSF-CCL18 loop plays a critical role in the progression of breast cancer (38). In another study, *in vitro* co-culture of human MDA-MB-231 breast cancer cells with mouse macrophages led to the upregulation of MCP-1 production by mouse macrophages (39); however, the mechanisms by which breast cancer cells upregulate MCP-1 production by macrophages remain uncharacterized.

In the present study, we first detected that a product(s) of 4T1 mouse breast cancer cells could upregulate MCP-1 production by macrophages. The majority of the MCP-1-inducing activity was retained after filtration through a membrane with a molecular weight cut off of 30 kDa, suggesting that the molecular mass of the 4T1 product might be larger than 30 kDa. However, the results of HPLC gel-filtration indicated that the molecular mass of the 4T1 product was approximately 20–26 kDa, significantly smaller than that obtained by the membrane. This 4T1 cell product was subsequently identified as GM-CSF using a neutralizing antibody against GM-CSF. The apparent molecular mass of GM-CSF purified from mouse lung-conditioned medium was approximately 29 kDa by gel-filtration and 23 kDa by electrophoresis (40). Thus, the molecular mass of the 4T1 product we identified as GM-CSF was similar to that of purified natural mouse GM-CSF. The membrane we used was characterized by a nominal molecular weight limit, but its retaining capacity may be affected by other factors, such as physicochemical characteristics and behavior in a particular solution of each protein. In fact, as reported by the manufacturer of the membrane, 70% of α-chymotrypsinogen whose molecular mass was 25 kDa was retained by the same membrane. This may explain the reason why the majority of the MCP-1-inducing activity was retained by this membrane despite that the molecular mass of GM-CSF is smaller than 30 kDa.

Granulocyte/macrophage colony-stimulating factor induces a high level of MCP-1 expression and/or production in both mouse and human macrophages at a concentration as low as 1 ng/ml. Interestingly, it induces only a modest level of KC or MIP-2 expression, and the expression level of these neutrophil chemotactic chemokines was much lower than that induced by the TLR4 ligand LPS. Furthermore, GM-CSF upregulates the expression of certain genes whose expression was not significantly upregulated by LPS, indicating that GM-CSF preferentially induce the expression of a unique set of genes, including MCP-1. Our results strongly suggest that in addition to the GM-CSF-CCL18 loop noted above, the GM-CSF-MCP-1 (CCL2) loop is also present in breast cancer. Both CCL18 and MCP-1 can promote breast cancer progression in human. Since CCL18 is absent in mice (41), it is plausible that MCP-1 may act as a chemokine counterpart of human CCL18 for breast cancer progression in mice. In contrast to the tumor-promoting activity of endogenously produced GM-CSF, exogenously administered GM-CSF elicits potent immune responses and thus is used in tumor therapies as an immune adjuvant (42). Therefore, it will be important to delineate how the administration of exogenous GM-CSF affects the GM-CSF-CCL18 or GM-CSF-MCP-1 loop created by endogenous GM-CSF. The availability of GM-CSF in different locations of cancer subjects may be critical to enhance the immune system.

Although tumor-cell-derived GM-CSF markedly upregulated MCP-1 production by macrophages *in vitro*, neutralization of GM-CSF in tumor-bearing mice showed little effect on serum MCP-1 concentration or tumor MCP-1 content, strongly suggesting that the GM-CSF is not a major regulator of MCP-1 production in 4T1 tumor microenvironment. Furthermore, there was no reduction in the number of metastatic lung tumors, the number of splenocytes, or the percentage of tumor-infiltrating MDSCs or macrophages in tumor-bearing mice treated with anti-GM-CSF Ab. These results indicate the presence of GM-CSF-independent mechanisms, which upregulate MCP-1 production in breast cancer tumor microenvironments. Macrophage production of MCP-1 can be upregulated by other molecules, including TLR ligands. This may explain why neutralization of GM-CSF did not result in the reduction of MCP-1 production as evidenced by similar serum MCP-1 levels in control and anti-GM-CSF Ab-treated mice. It is also well known that other cellular components of tumor stroma, such as fibroblasts or endothelial cells, produce MCP-1 in response to stimulation (43). Co-culture of human fibroblasts with human breast cancer cells tumor previously resulted in the production of MCP-1 by fibroblasts (44), suggesting that fibroblasts can be an important cellular source of MCP-1 in tumor microenvironments. We will explore this possibility in the future.

Unlike MCP-1^−/−^ mice in which lung metastasis, but not the size of tumors at the injected site, was reduced (16), anti-GM-CSF treatment significantly reduced tumor size without affecting the level of lung metastasis. In the present study, we found that GM-CSF activates macrophages and induces the expression of MCP-1. However, MCP-1 may not be the only cytokine whose expression is upregulated by GM-CSF in tumor microenvironments. Macrophages cultured with GM-CSF were previously demonstrated to become cells capable of producing TNFα or IL-6 following stimulation with the TLR4 ligand LPS (45, 46). In fact, we detected these cytokines in the culture supernatant of 4T1-sup-activated macrophages by ELISA and upregulation of mRNA expression by GM-CSF-activated macrophages by qRT-PCR. In addition, GM-CSF induced the upregulation of several other genes whose products could also potentially promote tumor growth. GM-CSF was also shown to stimulate granulocytes to produce prostaglandin E and leukotriene B4, which can dampen antitumor immune responses (45–47). Thus, GM-CSF produced by tumors cells can promote tumor growth by inducing the production of multiple tumor-promoting cytokines and chemokines by macrophages and by activating neutrophils. This may explain the reason why the neutralization of GM-CSF resulted in reduced 4T1 tumor growth. Further studies are necessary to identify the precise role of tumor cell-derived GM-CSF in tumor development.

A number of proinflammatory mediators are present in tumor microenvironments, which are produced by either tumor cells or stromal cells. Although in some tumor cells, the production of proinflammatory mediators is intrinsically upregulated, the interaction of tumor cells with stromal cells is a key factor for upregulated production of certain tumor-promoting proinflammatory mediators. We recently revealed that Lewis lung carcinoma cells activate mouse macrophages to produce TNFα which in turn activates Lewis lung carcinoma cells to produce a large amount of MCP-1 (48). Additional studies identifying the mechanisms of tumor cell-stromal cell interaction and molecule(s) responsible for the induction of tumor-promoting mediators are necessary to provide a new means to target tumor microenvironment.

## Conflict of Interest Statement

The authors declare that the research was conducted in the absence of any commercial or financial relationships that could be construed as a potential conflict of interest.
