# Corrigendum: Induction of Monocyte Chemoattractant Proteins in Macrophages *via* the Production of Granulocyte/Macrophage Colony-Stimulating Factor by Breast Cancer Cells

**DOI:** 10.3389/fimmu.2017.01524

**Published:** 2017-11-13

**Authors:** Teizo Yoshimura, Tomozumi Imamichi, Jonathan M. Weiss, Miwa Sato, Liangzhu Li, Akihiro Matsukawa, Ji Ming Wang

**Affiliations:** ^1^Cancer and Inflammation Program, Center for Cancer Research, National Cancer Institute, Frederick, MD, United States; ^2^Department of Pathology and Experimental Medicine, Graduate School of Medicine, Dentistry and Pharmaceutical Sciences, Okayama University, Okayama, Japan; ^3^Laboratory of Human Retrovirology and Immunoinformatics, Leidos Biomedical Research, Inc., Frederick National Laboratory for Cancer Research, Frederick, MD, United States; ^4^Engineering Research Center for Cell and Therapeutic Antibody of Ministry of Education, School of Pharmacy, Shanghai Jiaotong University, Shanghai, China

**Keywords:** monocytes/macrophages, chemokines, inflammation, tumor microenvironment, breast cancer

**Text Correction**

In the original article, there was an error. **In the abstract, we stated that the molecular mass of the 4T1 cell product was 2–3 kDa**.

A correction has been made to **Abstract**, **line 10, 20–30 kDa**.

The authors apologize for this error and state that this does not change the scientific conclusions of the article in any way.

## Conflict of Interest Statement

The authors declare that the research was conducted in the absence of any commercial or financial relationships that could be construed as a potential conflict of interest.

