# Supplementary material for: Induction of Monocyte Chemoattractant Proteins in Macrophages via the Production of Granulocyte/Macrophage Colony-Stimulating Factor by Breast Cancer Cells
Source: Front Immunol. 2016 Jan 20;7:2. doi: 10.3389/fimmu.2016.00002 (PMC4718995; doi:10.3389/fimmu.2016.00002)
Supplement: Supplementary file 1 [file Data_Sheet_1.DOCX]

Table 1. Relative expression of cytokine/chemokine genes by GM-CSF- or LPS-activated mouse peritoneal exudate macrophages

Gene name Relative expression

GM-CSF LPS

TNF (TNF superfamily, member 2) 25.7 117.0

Lymphotoxin A (TNF superfamily, member 1) 9.3 6.4

Lymphotoxin B (TNF superfamily, member 3) 1.8 1.1

TNF superfamily, member 4 3.6 2.6

CD40 ligand (TNF superfamily, member 5) 12.0 2.0

Fas ligand (TNF superfamily, member 6) 1.4 0.8

CD70 (TNF superfamily, member 7) 1.6 3.3

TNF superfamily, member 8 1.8 0.6

TNF superfamily, member 9 2.9 7.6

TNF superfamily, member 10 6.1 7.2

TNF superfamily, member 11 80.1 6.1

TNF superfamily, member 12 4.3 0.2

TNF superfamily, member 13b 1.3 0.4

TNF superfamily, member 14 12.8 0.8

TNF superfamily, member 15 0.5 14.6

TNF superfamily, member 18 8.5 0.6

Ectodysplasin-A nd nd

IL-1alpha 22.7 170.0

IL-1beta 19.0 127.0

IL-1Ra 19.7 0.1

IL-1 family, member 5 (delta) 0.8 0.5

IL-1 family, member 6 1.6 48.4

IL-1 family, member 8 47.4 0.1

IL-2 nd nd

IL-3 nd nd

IL-4 nd nd

IL-5 1.4 3.5

IL-6 2486.5 68659.5

IL-7 nd nd

IL-9 nd nd

IL-10 1.9 1136.1

IL-11 nd nd

IL-12a 1.3 27.6

IL-12b nd nd

IL-13 nd nd

IL-15 1.4 8.6

IL-17B nd 0.5

IL-17C 1.5 0.2

IL-17D 4.1 0.3

IL-17F 25.3 618.7

IL-18 0.8 3.6

IL-19 0.2 465.1

IL-20 nd nd

IL-21 nd nd

IL-23, p19 nd nd

IL-24 nd nd

IL-28 nd nd

CXCL1 64.7 1860.7

CXCL2 94.7 418.2

CXCL3 34.5 20.0

CXCL5 255.7 3537.5

CXCL7 4.9 0.6

CXCL9 0.6 5.2

CXCL10 3.2 16.3

CXCL11 5.2 25.1

CXCL12 5.0 418.2

CXCL13 9.5 20.0

CXCL14 2.7 0.1

CXCL16 2.0 2.2

CCL2 35.6 7.5

CCL3 41.7 47.3

CCL4 8.9 34.3

CCL5 1.3 50.1

CCL7 86.2 16.0

CCL8 1.0 0.4

CCL11 47.7 25.1

CCL17 229.2 1.0

CCL19 nd nd

CCL20 1.9 1.4

CCL21a 0.1 1.1

CCL22 18.7 13.1

CCL24 33.4 3.0

CCL25 0.7 0.2

CCL27 0.4 0.3

CCL28 8.0 2.2

The expression of each gene was examined by qPCR using primer libraries purchased from RealTimePrimers.com. Some of the genes preferentially expressed by GM-CSF- or LPS-activated macrophages are underlined.
